# Identification of a Novel PROP1 Mutation in a Patient with Combined Pituitary Hormone Deficiency and Enlarged Pituitary

**DOI:** 10.3390/ijms20081875

**Published:** 2019-04-16

**Authors:** Laura Penta, Carla Bizzarri, Michela Panichi, Antonio Novelli, Francesca Romana Lepri, Marco Cappa, Susanna Esposito

**Affiliations:** 1 Pediatric Clinic, Department of Surgical and Biomedical Sciences, Università degli Studi di Perugia, Piazza Lucio Severi 1, 06132 Perugia, Italy; laura.penta@ospedale.perugia.it; 2 Unit of Endocrinology and Diabetes, IRCCS Bambino Gesù Children’s Hospital, Piazza Sant’Onofrio 4, 00165 Rome, Italy; Carla.bizzarri@opbg.net (C.B.); marco.cappa@opbg.net (M.C.); 3 Unit of Pediatrics, Città di Castello Hospital, Via L. Angelini, 10, 06012 Città di Castello, Italy; michela.panichi@uslumbria1.it; 4 Laboratory of Medical Genetics, IRCCS Bambino Gesù Children Hospital and Research Institute, Piazza Sant’Onofrio 4, 00165 Rome, Italy; antonio.novelli@opbg.net (A.N.); francescaromana.lepri@opbg.net (F.R.L.)

**Keywords:** combined pituitary hormone deficiency, growth hormone deficiency, isolated growth hormone deficiency, pituitary gland, PROP1

## Abstract

Growth hormone deficiency (GHD) can be present from the neonatal period to adulthood and can be the result of congenital or acquired insults. In addition, GHD can be classified into two types: isolated growth hormone deficiency (IGHD) and combined pituitary hormone deficiency (CPHD). CPHD is a disorder characterized by impaired production of two or more anterior and/or posterior pituitary hormones. Many genes implicated in CPHD remain to be identified. Better genetic characterization will provide more information about the disorder and result in important genetic counselling because a number of patients with hypopituitarism represent familial cases. To date, PROP1 mutations represent the most common known genetic cause of CPHD both in sporadic and familial cases. We report a novel mutation in the PROP1 gene in an infant with CPHD and an enlarged pituitary gland. Close long-term follow-up will reveal other possible hormonal defects and pituitary involution.

## 1. Introduction

Growth hormone deficiency (GHD) can be present from the neonatal period to adulthood and can be the result of congenital or acquired insults. GHD is classified into two major types: isolated growth hormone deficiency (IGHD) and combined pituitary hormone deficiency (CPHD) [1]. CPHD is a disorder characterized by impaired production of two or more anterior and/or posterior pituitary hormones. CPHD clinical features may include short stature, hypothyroidism, impaired sexual maturation, and hypo-cortisolism, either individually or simultaneously [2]. CPHD can present with or without extra-pituitary features such as optic nerve hypoplasia and midline forebrain defects. CPHD may result from acquired lesions in the hypothalamic-pituitary area (tumor, trauma, surgery, or irradiation) or from genetically defined conditions [3]. In some patients with CPHD, the disorder is associated with mutations in genes involved in anterior pituitary development. The condition may be sporadic or familial, with familial cases accounting for 5% to 30% of cases [4].

The pituitary function depends on the integrity of the hypothalamic-pituitary axis and any defect in the development or organogenesis of the gland may cause hormonal deficiencies [5]. Embryonic development follows a well-defined pattern of sequential expression of pituitary-specific transcription factors such as Prophet of PIT-1 (PROP1), PIT-1 (POU1FI), HESX-1, SOX2, SOX3, LHX-3, and LHX-4 [1]. Mutations in any of the genes encoding these transcription factors can lead to congenital hypopituitarism, which is often associated with a wide spectrum of defects affecting craniofacial/midline development [6]. PROP1 mutations are implicated in non-syndromic CPHD, including progressive growth hormone (GH), thyrotropin (TSH), and prolactin (PRL) defects, in addition to a variable defect in luteinizing hormone (LH)/follicle-stimulating hormone (FSH) and adrenocorticotropic hormone (ACTH) secretion. In younger children, IGHD can evolve in CPHD over time [4].

To date, PROP1 mutations represent the most common known genetic cause of CPHD both in sporadic (6.7%) and familial cases (48.5%), with a global mutation frequency of 11% considering all patients [1]. The mutation rate varies considerably among geographic areas. Two variants, namely, c.301_302delAG and c.150delA are the most common PROP1 mutations and represent more than 90% of the mutated alleles in the Eastern European cohorts [7].

We report a novel mutation in the PROP1 gene in an infant with CPHD and an enlarged pituitary.

## 2. Case Presentation

Our case is a Caucasian Italian female infant who is the second child of healthy, unrelated parents. She was born at 41 weeks of gestational age by normal delivery with a birth weight of 3.222 kg (weight −0.72 standard deviation score [SDS]) and a birth length of 48 cm (−1.57 SDS). At birth, she presented mild jaundice, while blood glucose was normal. Decreased linear growth was first observed at the age of 3 years, without dysmorphic features or neurodevelopmental delay. At 3 years, the infant’s height was 78.7 cm (−4.23 SDS) and weight was 10.8 kg (−2.60 SDS). 

The father’s height was 164 cm, and the mother’s height was 160 cm. Thus, the mid parental height is 156 cm (−1.13 SDS) [8]. The paternal grandmother height was 150 cm and her mother’s height was 145 cm.

The baby presented troncular obesity and a doll face with a broad forehead. Bone age was approximately 1 year and 2 months [9]. The chemiluminescence immunoassay (IMMULITE-2000 XP analyzer, Siemens Healthcare Diagnostic Products, Milan, Italy) measured all laboratory tests with the help of commercial kits. Exams at admission showed normal findings except for low free thyroxine (FT4) levels (0.45 ng/dL, with references values [r.v.], 0.60–1.12), TSH: 2.410 µUI/mL (r.v., 0.340–5.600). Antithyreoglobulin and antithyroid peroxidase antibodies were absent. Again, cortisol levels were normal, both basal and after adrenocorticotrophic hormone [ACTH] stimulation), prolactine (PRL) was in the normal range and gonadotropins were low, according to Tanner stage 1. Both insulin-like growth factor 1 (IGF-1) and insulin-like growth factor binding protein 3 (IGFBP3) were within the normal values and a blunted peak GH response to the growth hormone releasing hormone (GHRH) plus arginine was obtained, with a maximum peak of 5.60 ng/mL (Table 1). Mean intra-assay and inter-assay variability coefficients were 4.8% and 5% for GH, 2.7% and 3.2% for TSH, 1.8% and 2.6% for FT4, 1.7% and 4.7% for cortisol, 3.2% and 8.2% for PRL, 4.9% and 5% for IGF-I, and 4.1% and 8% for IGFBP-3.

**Figure 1:**
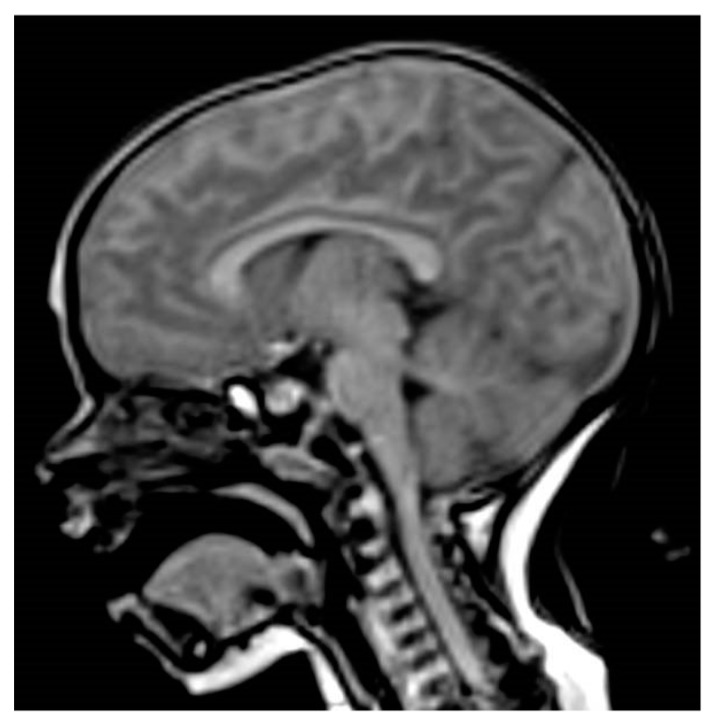
Sagittal pituitary MRI image showing hyper-intense enlarged anterior pituitary (T1-weighted sequence).

Pituitary magnetic resonance imaging (MRI) showed a T1 hyper-intense enlarged anterior pituitary (height 9 mm), symmetrical region with a patchy signal and low enhancement after contrast agent (gadolinium) injection, with no abnormalities in the pituitary stalk or the optic chiasm, and a normal posterior pituitary bright spot (Figure 1). Brain MRI was performed using a 3 Tesla magnet (Samsung, Seregno [MB], Italy). Sequences were acquired according to multiple planes and a thin layer on the diencephalon-pituitary region. The sequences were obtained before and after the intravenous administration of paramagnetic contrast medium (Dotarem 0.5 mmol/mL, tot 2.6 mL) with suppression of the adipose tissue signal.

Pituitary enlargement with CPHD (i.e., impaired production of two pituitary hormones [TSH and GH]) led to mutational analysis of the PROP 1 gene.

**Figure 2:**
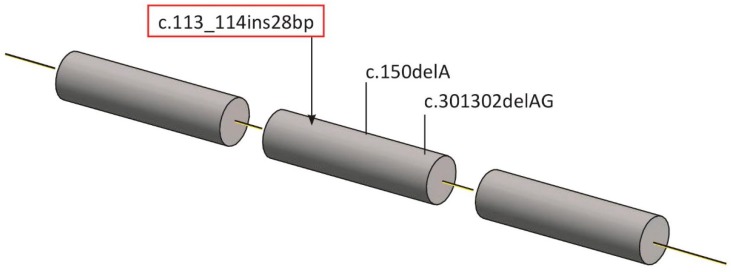
Scheme describing the PROP1 gene with points of the common mutations (301_302delAG and c. 150delA) and the region of the novel identified mutation.

Genomic DNA was isolated from peripheral blood leukocytes according to standard methods [10], using a commercial kit (Macherey-Nagel, Düren, Germany). Sanger Sequencing (GenBank accession number NM_006261) analyzed the entire coding sequence, exon/intron boundaries, and flanking intronic portions of the PROP1 gene. Primer pair sequences and sequencing analysis settings are available upon request. Sequencing analysis allowed the identification of a homozygous mutation c.113_114ins28bp in exon 2 of the PROP1 gene in the proband (Figure 2). Both parents were heterozygous for these mutations.

**Figure 3:**
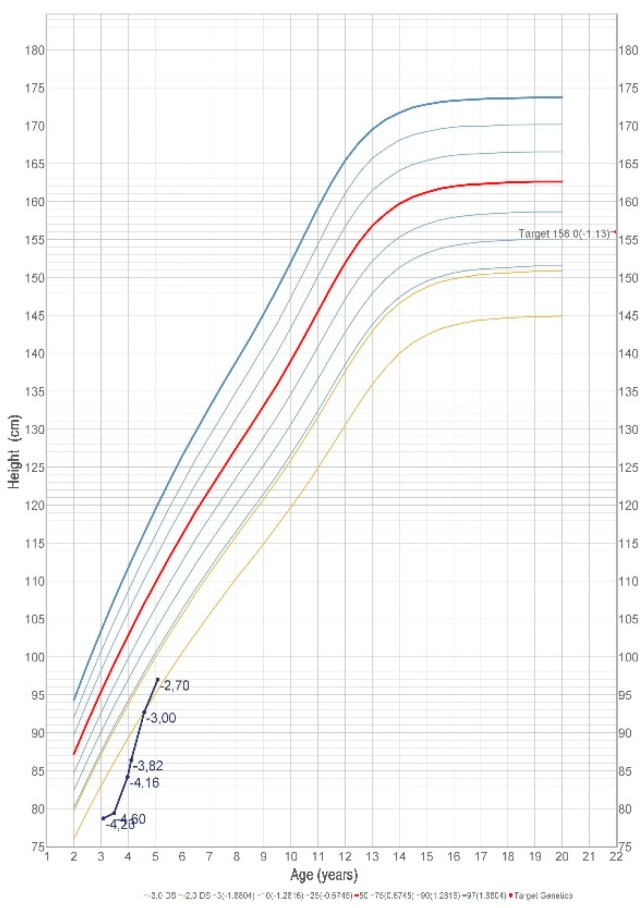
Increase in growth after 18 months of GH and LT4 therapy.

**Figure 4:**
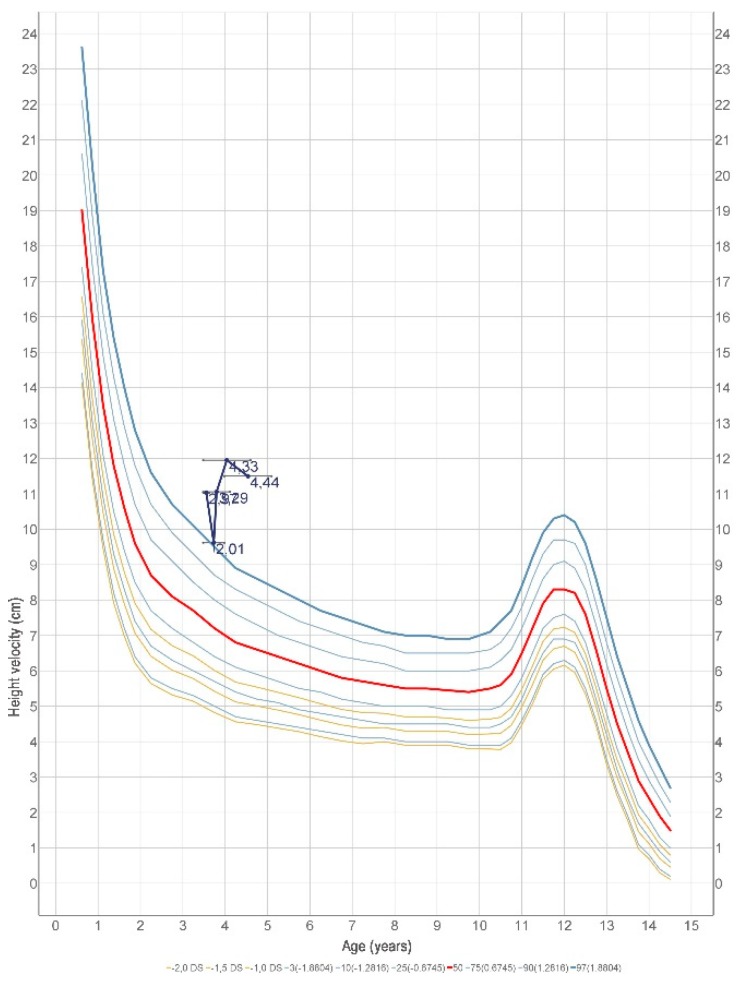
Growth velocity over the same period.

Concerning the presence of GH and thyroxine deficit, a replacement therapy was started using recombinant human growth hormone (hGH 0.028 mg/kg/day, 0.67 mg/m^2^/day) and levotiroxine LT4 (1.77 mg/kg/day). After 11 months of therapy, the height was 92.7 cm (−3 SDS), and the growth velocity increased to 11.95 cm/year. After 18 months, the height became 97 cm (−2.70 SDS), which indicates a good response to GH therapy with height velocity, SDS +4.33 SDS (Figure 3 and Figure 4). No side effects were reported. We assessed hormonal normalization of IGF1 and L-T4, after replacement therapy. After 11 months of therapy, IGF-1 (102 ng/mL) and also IGFBP-3 (4.7 µg/mL) and FT4 (0.85 ng/dL) showed normal values. After 18 months of therapy, IGF-1 and FT4 were still in normal values, respectively, which were 98.9 ng/mL and 1.04 ng/dL. We decided to not perform a new pituitary MRI because a good response to the therapy allowed us to delay this control.

Clinical information and blood samples were obtained after approval from the Ethics Committee of the Umbria Region (PED-2018-21) and written informed consent was obtained from both parents. Parents also signed the consent for the publication of this case report.

## 3. Discussion

Mutations in genes encoding transcription factors can lead to congenital hypopituitarism, which is often associated with a wide spectrum of defects affecting craniofacial/midline development [6]. PROP1 is a transcription factor involved in pituitary gland development. PROP1 mutations constitute the most common genetic cause of CPHD. Mutations in PIT1 (POUF1) and PROP1 gene—“later-acting transcription factors” in pituitary organogenesis—are responsible for a pituitary phenotype with multiple hormone deficiencies without extra-pituitary findings, whereas mutations such as HESX1 and GLI2—“early transcription factors”—may present extra-pituitary manifestations, including syndromic hypopituitarism with septo-optic dysplasia, holoprosencephaly, or other craniofacial defects [7]. In humans, the PROP1 gene is located on chromosome 5q and encodes a protein composed of 226 amino acids. The two most common mutations are a two-base-pair deletion in codon 101 (301–302 delAG) and a one-base-pair deletion in codon 150 (150 del A). However, many other missense, nonsense, and splice error mutations have been described [11,12]. We report a novel mutation in the PROP1 gene in a three-year-old infant with CPHD and an enlarged pituitary. The mutation we found is a novel one that most likely produces impaired translation of the protein product.

The transcription factor PROP1 is a key regulator of the differentiation of pluripotent anterior pituitary cells into distinct cell lines with characteristic secretory profiles. PROP1 is an acronym for Prophet of PIT. The transcription factor precedes and is required for the expression of PIT1, which is another transcription factor expressed during pituitary development [12]. A mutation in PROP1 results in failure to express PIT1 protein and to develop somatotrophic, lactotrophic, and thyrotrophic cells. The cell lines specifically regulated by PROP1 are somatotrophs, lactotrophs, and thyrotrophs. Involvement of PROP1 in the differentiation of gonadotrophs and corticotroph is more variable [13]. Hence, PROP1 mutations are implicated in non-syndromic CPHD with a phenotype characterized by deficiencies of GH, PRL, TSH, LH, and FSH and later-onset ACTH deficiency. Generally, hormone deficiencies present a typical temporal pattern: first GH and TSH and then FSH, LH, and ACTH. The degree of PRL deficiency is variable [12]. Therefore, pituitary function monitoring in patients with PROP1 mutations is mandatory.

CPHD due to PROP1 mutations can present different patterns of evolution [2]. Many patients with PROP1 defects have growth failure in late infancy or childhood with no neonatal sign of GH deficiency, such as hypoglycaemia, seizures, and prolonged jaundice [12]. Our patient presented a non-syndromic short stature without neonatal signs of a GH deficit.

All patients with PIT1 mutations and most patients with PROP1 mutations have either small or normal-sized anterior pituitary glands, but some patients carrying PROP1 mutations can present normal-sized or hypoplastic or even enlarged pituitary glands. Several patients with PROP1 mutations and pituitary enlargement are usually observed in early childhood, which is followed by pituitary hypoplasia [14]. The 301,302delAG mutation is the most frequent mutation and most patients with pituitary enlargement have been shown to harbor the 301–302delAG mutation. However, 150delA, Q83X, and R73C mutations have also been described as associated with pituitary enlargement. Usually, pituitary enlargement is followed by a waxing and waning size of the pituitary mass with hypoplastic pituitary at the end. In addition, the same mutation in siblings can occur with different pituitary morphologies [14]. Our case underlines the importance of MRI imaging in the diagnosis of children with hypopituitarism [6]. Differences in MRI pituitary gland morphology can suggest different GHD etiologies and prognoses. In our patient, the finding of an enlarged pituitary in a non-syndromic infant with two pituitary hormone deficiencies suggested a PROP 1 defect. Pituitary enlargement is sometimes dramatic but has a benign course and is followed by degeneration and regression [12]. Under such conditions, genetic testing offers an important tool for making therapeutic decisions in patients with pituitary mass and is indispensable in avoiding unnecessary neurosurgery [13].

Identifying the exact genotype, in addition to the endocrine and MRI phenotype, could be very helpful in the management of patients with GHD because an early diagnosis of evolving anterior pituitary hormone deficiencies will be possible. For example, a patient with an identified PROP1 mutation should be carefully monitored for an evolving hypopituitarism, while a patient carrying a mutation in PIT1 would most likely present normal cortisol and gonadotrophin secretion levels [6].

## 4. Conclusions

We identified a novel mutation of the PROP1 gene associated with GH and TSH defects and associated with an enlarged pituitary gland. Close long-term follow-up will reveal other possible hormonal defects and pituitary involution. Further studies should evaluate genes implicated in CPHD, and better genetic characterization will provide further information about the disorder, which is also important for genetic counselling because a number of patients with hypopituitarism represent familial cases.

## Figures and Tables

**Table 1:** Hormonal evaluation of anterior pituitary function in the affected child. GH: growth hormone. IGF1: insulin-like growth factor 1. TSH: thyroid stimulating hormone. PRL: prolactine. The immuno-chemiluminometric assay measured all hormones.

Data	Age	IGF-1	IGFBP3	GH Basal	GH Peak Response after GHRH + Arginine	Cortisol Basal	Cortisol after ACTH 1 μg	TSH	FT4	PRL
	Years	ng/mL	μg/mL	ng/mL	ng/mL	μg/dL	μg/dL	µUI/mL	ng/dL	ng/mL
Results	3	<25.0	<0.6	0.55	5.60	12.46	22.69	2.410	0.45	18.9
